# Preoperative Tumor-Informed ctDNA for Prediction of Nodal Metastases at Radical Cystectomy in Bladder Cancer

**DOI:** 10.1245/s10434-026-20022-7

**Published:** 2026-06-21

**Authors:** Can Aydogdu, Betty Wang, Sean McSweeney, Alberto Pieretti, Christopher J. Weight, Riccardo Autorino, Jihad Kaouk, Laura Bukavina

**Affiliations:** 1 Department of Urology, Integrated Surgical Institute, Cleveland Clinic, Cleveland, OH USA; 2 Department of Urology, LMU University Hospital Munich, Munich, Germany; 3 Department of Urology, Weston Hospital, Cleveland Clinic Florida, Weston, FL USA; 4 Cleveland Clinic Lerner College of Medicine, Cleveland Clinic, Cleveland, OH USA

**Keywords:** Bladder cancer, Radical cystectomy, Pelvic lymph node dissection, Circulating tumor DNA, Molecular residual disease, Nodal metastasis, Risk stratification, Precision surgery

## Abstract

**Background:**

This study aimed to evaluate whether pre-surgical tumor-informed circulating tumor DNA (ctDNA) predicts pathologic nodal status at radical cystectomy and its potential role in risk stratification for future biomarker-guided surgical strategies.

**Methods:**

The study prospectively analyzed 40 patients with non–muscle-invasive and muscle-invasive bladder cancer who underwent radical cystectomy with pelvic lymph node dissection (LND) and pre-surgical tumor-informed ctDNA testing. Compared with pathologic nodal findings, ctDNA status was classified as positive or negative. Diagnostic performance metrics with 95 % confidence intervals (CIs) were calculated. To assess reproducibility, data from a published cohort meeting comparable inclusion criteria were integrated for pooled descriptive analysis.

**Results:**

Of 40 patients, 27 (68 %) were ctDNA-negative and 13 (32 %) were ctDNA-positive before cystectomy. Pathologic nodal metastases were identified in seven patients (18 %). For nodal metastases, ctDNA demonstrated a sensitivity of 86 % (95 % CI, 49–97 %), a specificity of 79 % (95 % CI, 62–89 %), and negative predictive value of 96 % (95 % CI, 82–99 %). In pooled descriptive analysis including 149 patients, the negative predictive value remained high at 92 % (95 % CI, 84–97 %). A simulated ctDNA-guided omission strategy may have avoided LND for 27 patients (68 %), with one false-negative case (3.7 %) that subsequently had recurrence.

**Conclusion:**

Pre-surgical tumor-informed ctDNA showed high negative predictive value for nodal metastases at radical cystectomy. These findings supported prospective validation of ctDNA as a biomarker to identify patients at low risk of nodal metastases and inform future trials of biomarker-guided surgical strategies.

**Supplementary Information:**

The online version contains supplementary material available at https://doi.org/10.1245/s10434-026-20022-7.

Pelvic lymph node dissection (LND) at the time of radical cystectomy remains a standard component of surgical management for bladder cancer, providing essential staging information and contributing to local disease control.^1,2^ However, the extent of dissection and its incremental therapeutic benefit beyond standard templates remain areas of ongoing investigation.

Although LND provides important staging information, randomized trials have not demonstrated a clear survival advantage for more extensive templates. The phase 3 LEA AUO AB 25/02 trial showed no survival benefit for super-extended versus standard dissection and reported higher major complication rates.^3^ Similarly, the SWOG S1011 trial failed to demonstrate an overall survival advantage for extended LND and was associated with increased 90-day perioperative mortality (6.5 % vs 2.7 %). These findings suggest that more extensive LND does not confer additional survival benefit compared with standard templates while being associated with increased perioperative risk.

At the same time, pelvic LND is associated with measurable morbidity, including lymphocele formation, lymphedema, rare vascular injury, and additional operative time. Despite these risks, patient selection remains imprecise because conventional imaging lacks sufficient sensitivity for detecting nodal metastases.^4^ A reliable preoperative biomarker capable of identifying patients at low risk for nodal involvement could therefore refine surgical decision-making and reduce unnecessary procedures.

Circulating tumor DNA (ctDNA) has emerged as a sensitive marker of minimal residual disease and systemic tumor burden across malignancies. In urothelial carcinoma, tumor-informed ctDNA assays have demonstrated prognostic value and the ability to detect molecular disease beyond conventional imaging, including detection in non–muscle-invasive settings.^5–8^ However, whether preoperative ctDNA can identify patients unlikely to harbor nodal metastases and thereby inform risk-adapted surgical strategies has not been systematically evaluated.

We therefore investigated whether pre-surgical tumor-informed ctDNA predicts pathologic nodal status at radical cystectomy and whether ctDNA may identify a subset of patients at low risk for nodal involvement. We hypothesized that ctDNA-negative status would be associated with a low likelihood of nodal metastases and could inform the design of prospective studies evaluating biomarker-guided refinement of pelvic LND.

## Patients and Methods

### Patients

This institutional cohort study included patients with non–muscle-invasive and muscle-invasive urothelial carcinoma who underwent radical cystectomy with pelvic LND between 2024 and 2025. Blood samples for tumor-informed ctDNA analysis were collected prospectively as part of clinical care, and subsequent data analysis was performed retrospectively. Inclusion required availability of preoperative ctDNA testing and complete pathologic nodal staging. All patients were clinically node-negative (cN0) based on preoperative imaging.

The study was approved by the institutional review board (IRB no. 24-921) and conducted in accordance with the Declaration of Helsinki. The requirement for informed consent for this analysis was waived by the institutional review board.

All the patients underwent radical cystectomy with pelvic LND according to a standardized institutional template including bilateral external iliac, internal iliac, and obturator nodal basins, performed using an open or robotic approach. Surgical specimens were processed according to institutional protocols and staged per the eighth edition of the American Joint Committee on Cancer (AJCC) tumor-node-metastasis (TNM) classification by dedicated genitourinary pathologists.

### Endpoints

The primary endpoint was the negative predictive value of preoperative tumor-informed ctDNA for pathologic nodal metastases at radical cystectomy. Pathologic nodal status was categorized as negative (pN0) or positive (pN1–3). The secondary endpoints included sensitivity, specificity, positive predictive value, overall accuracy, odds ratio, and area under the receiver operating characteristic curve.

To assess reproducibility, published data from Ben-David et al.^9^ were incorporated into a pooled descriptive analysis with the current cohort. That study included patients with urothelial carcinoma treated with radical cystectomy and pelvic LND who underwent preoperative tumor-informed ctDNA testing using the same Signatera assay platform (Signatera; Natera Inc., Austin, TX, USA) and had complete pathologic nodal staging. The definitions of ctDNA positivity and nodal status were identical to those used in the current study. The study excluded patients without available pathologic nodal status. Combined diagnostic performance estimates were calculated from aggregated 2×2 contingency tables constructed from both cohorts as a descriptive assessment of directional consistency rather than formal external validation.

For the external cohort, only aggregated ctDNA-stratified data were available, and patient-level data were not accessible. Therefore, adjustment for between-study heterogeneity, differences in patient characteristics, or treatment patterns was not possible.

### Tumor-Informed ctDNA Assay

Tumor-informed ctDNA testing was performed using a personalized, multiplex polymerase chain reaction (PCR)-based ultra-deep sequencing assay (Signatera assay platform) based on trans-urethral resection of bladder tumor (TURBT) tissue. The study selected 16 patient-specific somatic single-nucleotide variants from whole-exome sequencing of matched tumor and germline DNA. Plasma cell-free DNA was extracted from 20 mL of peripheral blood and sequenced at a median depth greater than ×100,000. Samples were defined as ctDNA-positive if two or more tumor-specific variants were detected above background noise, corresponding to a detection limit of approximately 0.01 % variant allele frequency. All laboratory procedures were Clinical Laboratory Improvement Amendments of 1988 (CLIA)-certified and College of American Pathologists (CAP)-accredited.

All ctDNA samples were obtained preoperatively. Sampling occurred before chemotherapy (*n* = 3), during chemotherapy (*n* = 10), after chemotherapy (*n* = 12), or among patients who did not receive chemotherapy (*n* = 15). For analysis, the sample closest to radical cystectomy was used.

### Statistical Analysis

Sensitivity, specificity, positive predictive value, negative predictive value, and accuracy were calculated with 95 % confidence intervals (CIs) using the Wilson score method. Odds ratios with 95 % confidence intervals were derived from 2×2 contingency tables using the log-transformed Wald method. The area under the receiver operating characteristic (ROC) curve was estimated using the trapezoidal rule. Median follow-up data were calculated using the reverse Kaplan–Meier method. A false-negative rate of 5–10 % was defined a priori as clinically acceptable for consideration of LND omission. All analyses were performed in R version 4.3.2 (R Foundation for Statistical Computing, Vienna, Austria).

## Results

This study analyzed 40 consecutive patients who underwent radical cystectomy with pelvic LND and preoperative tumor-informed ctDNA testing. The median age was 70 years (interquartile range [IQR], 65–77 years), and 31 (78 %) of the 40 patients were male. Of the 40 patients, 32 (80 %) had muscle-invasive bladder cancer, and 8 (20 %) had non–muscle-invasive disease at diagnosis, all of whom had cT1 tumors. Among the patients with muscle-invasive disease, 17 (53 %) had organ-confined (cT2) disease, and 15 (47 %) had locally advanced (≥cT3) tumors.

Neoadjuvant therapy was administered to 25 patients (63 %), all within the muscle-invasive subgroup. The median follow-up period was 7.2 months (IQR, 4.2–9.5 months), and the median interval from ctDNA testing to surgery was 36 days (IQR, 19–57 days). Detailed baseline characteristics are summarized in Table 1. The median lymph node yield was 15 (IQR, 11–19), consistent with adequate nodal sampling.

**Table 1:** Clinical and demographic characteristics of the institutional cohort, including patients with non–muscle-invasive and muscle-invasive bladder cancer who underwent radical cystectomy with pre-surgical ctDNA testing

Characteristic	CCF–NMIBC *n* (%)	CCF–MIBC *n* (%)	CCF–combined *n* (%)
*n* (%)	8 (20)	32 (80)	40 (100)
Median age: years (IQR)	73 (69–74)	69 (66–77)	70 (65–77)
*Sex*			
Male	7 (88)	24 (75)	31 (78)
Female	1 (12)	8 (25)	9 (22)
Median BMI: kg/m^2^ (IQR)	26.2 (23.8–28.6)	28.5 (22.9–31.1)	28.2 (23.1–31.0)
*Race*			
White	6 (75)	28 (88)	34 (85)
Black	0 (0)	1 (3)	1 (2)
Unavailable	2 (25)	3 (9)	5 (13)
*Smoking status*			
Former	6 (76)	13 (41)	19 (48)
Current	1 (12)	8 (25)	9 (23)
Never	1 (12)	11 (34)	12 (30)
*cT stage at diagnosis*			
1	8 (100)	–	8 (20)
2	–	17 (53)	17 (43)
3	–	14 (44)	14 (35)
4	–	1 (3)	1 (2)
cN stage			
cN0	8 (100)	32 (100)	40 (100)
*Neoadjuvant therapy*			
Yes	–	25 (78)	25 (63)
No	8 (100)	7 (22)	15 (37)
*ctDNA result*			
Negative	6 (75)	21 (66)	27 (68)
Positive	2 (25)	11 (34)	13 (32)
pT0N0			
ctDNA-negative	2 (25)	13 (41)	15 (38)
ctDNA-positive	0 (0)	1 (3)	1 (2)
*Lymph node metastases*			
No	7 (88)	26 (81)	33 (82)
Yes	1 (12)	6 (19)	7 (18)
Median lymph node yield (IQR)	19 (14–20)	13 (11–19)	15 (11–19)

ctDNA, circulating tumor DNA; CCF, Cleveland Clinic Foundation; NMIBC, non–muscle‐invasive bladder cancer; MIBC, muscle‐invasive bladder cancer; IQR, interquartile range; BMI, body mass index; cT, clinical T stage; cN, clinical N stage; CCF, Cleveland Clinic cohort

**Fig. 1:**
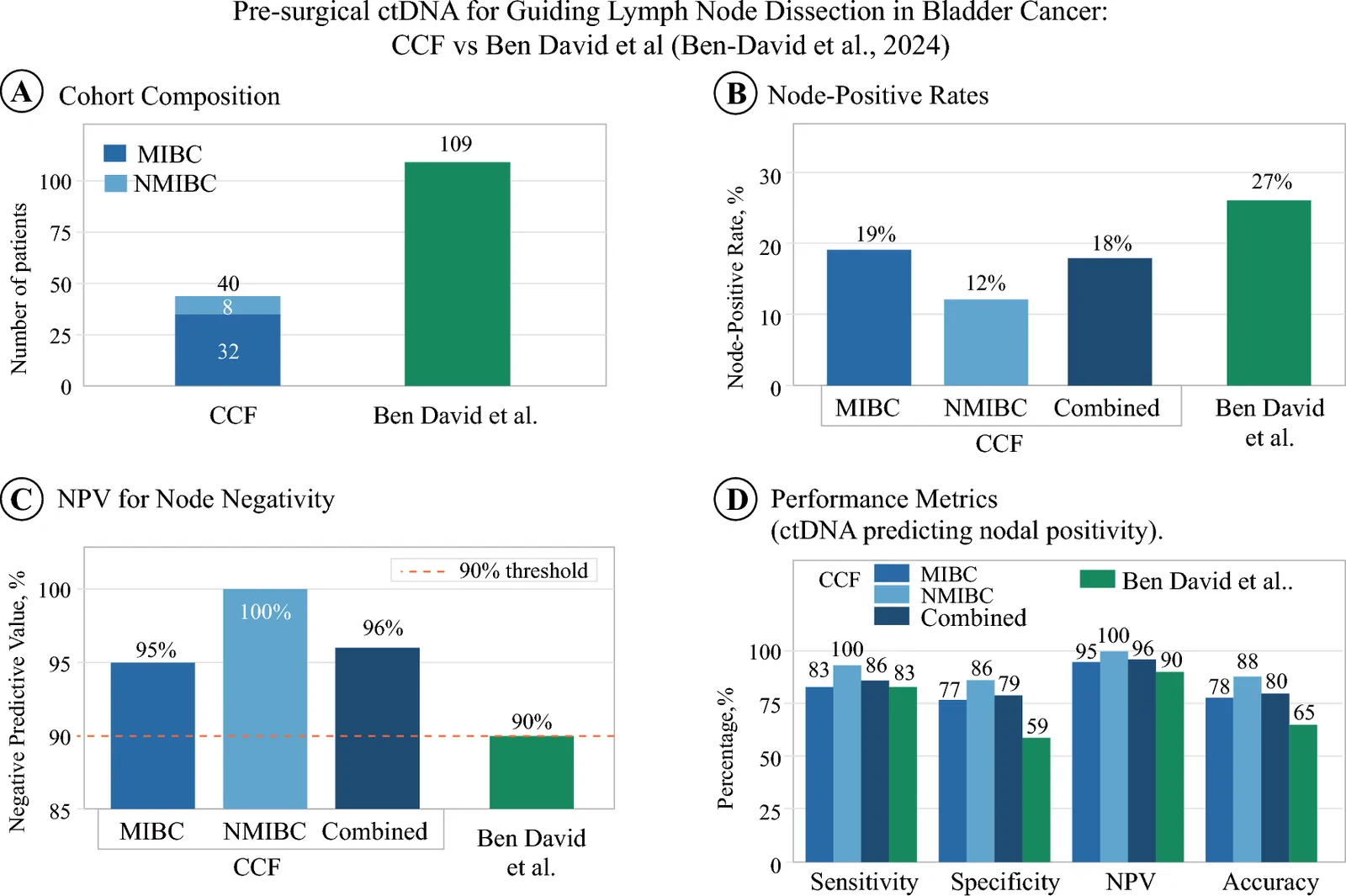
Cohort characteristics and diagnostic performance of ctDNA for prediction of nodal positivity. **A** Cohort composition showing the distribution of MIBC and NMIBC cases in the CCF cohort compared with the cohort reported by Ben David et al. **B** Node‐positive rates in MIBC, NMIBC, and the combined CCF cohort, with comparison to Ben David et al. **C** Negative predictive value for nodal negativity in the CCF subgroups and combined cohort, benchmarked against a 90 % threshold and compared with Ben David et al. **D** Performance metrics of ctDNA for predicting nodal positivity, including sensitivity, specificity, negative predictive value, and overall accuracy, shown for MIBC, NMIBC and the combined CCF cohort, with comparison to Ben David et al. ctDNA, circulating tumor DNA; MIBC, muscle‐invasive bladder cancer; NMIBC, non–muscle‐invasive bladder cancer; CCF, Cleveland Clinic Foundation; NPV, negative predictive value

Preoperatively, 27 patients (68 %) were ctDNA-negative, and 13 patients (32 %) were ctDNA-positive. Pathologic nodal metastases (pN1–3) were identified in seven patients (18 %). Pathologic complete response (pT0N0) was observed in 16 patients (40 %), including 15 who were ctDNA-negative and 1 who was ctDNA-positive (Fig. 1A and B).

Stratification by clinical stage showed higher ctDNA positivity in the ≥cT3 patients than in the cT2 patients (46.7 % vs 17.6 %; *p* = 0.18), whereas nodal positivity rates were similar between the groups (20 % vs 17.6 %; *p* = 1.00). These differences were not statistically significant and should be interpreted cautiously given the small sample size.

### Diagnostic Performance

In the institutional cohort, preoperative ctDNA demonstrated a negative predictive value of 96 % (95 % CI, 82–99 %) for pathologic nodal metastases. It had a sensitivity of 86 % (95 % CI, 49–97 %), a specificity of 79 % (95 % CI, 62–89 %), a positive predictive value of 46 % (95 % CI, 23–71 %), and an overall accuracy of 80 % (95 % CI, 65–90 %).

**Fig. 2:**
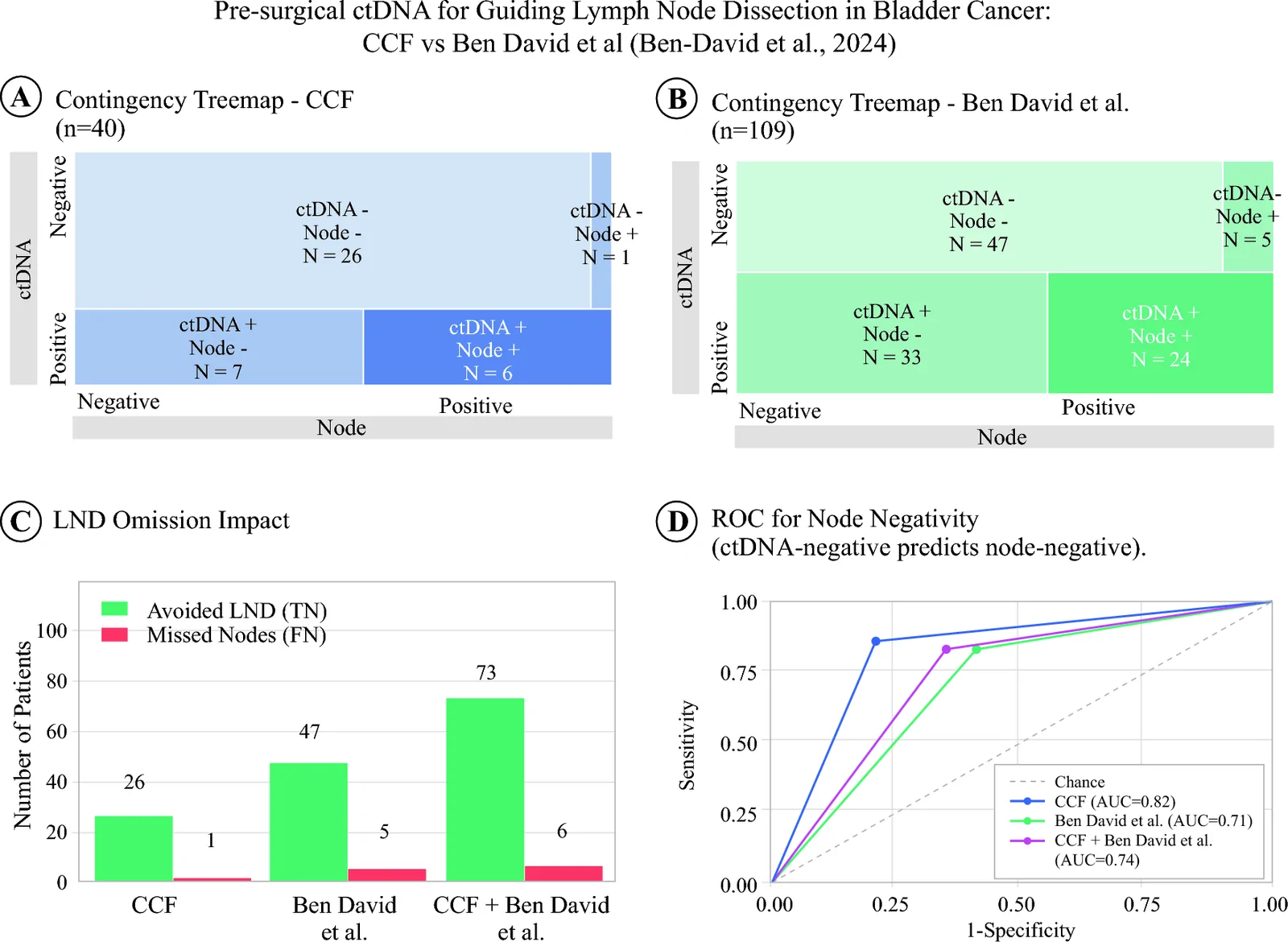
Pre‐surgical ctDNA and nodal status prediction in bladder cancer. **A** Contingency treemap for the CCF cohort (*n* = 40) showing ctDNA status according to pathologic nodal status. **B** Contingency treemap for the cohort reported by Ben David et al. (2024) (*n* = 109) showing ctDNA status by nodal status. **C** Estimated impact of ctDNA‐guided omission of LND demonstrating avoided LNDs (true-negatives) and missed node‐positive cases (false-negatives) in each cohort and pooled analysis. **D** Receiver operating characteristic curves for prediction of nodal negativity using ctDNA‐negative status, with corresponding area under the curve values for each cohort and the combined analysis. ctDNA, circulating tumor DNA; CCF, Cleveland Clinic Foundation; LND, lymph node dissection

The odds ratio for nodal metastases in ctDNA-positive patients was 22.29 (95 % CI, 2.29–216.93). The area under the receiver operating characteristic curve was 0.82. Receiver operating characteristic curves for each cohort are shown in Fig. 2D.

Stratified by disease stage, the negative predictive value was 95 % (95 % CI, 77–99 %) for the patients with muscle-invasive bladder cancer and 100 % (95 % CI, 61–100 %) for those with non–muscle-invasive disease (Table 2, Fig. 1C).

**Table 2:** Comparative diagnostic accuracy of ctDNA testing for nodal metastases in bladder cancer, stratified by study cohort^a^

Cohort	Sensitivity (N+) % (95 % CI)	Specificity (N+) % (95 % CI)	PPV % (95 % CI)	NPV % (95 % CI)	Accuracy % (95 % CI)	OR % (95 % CI)	AUC (N–)
CCF-MIBC	83 (44–97)	77 (58–89)	46 (21–72)	95 (77–99)	78 (61–89)	16.67 (1.62–171.8)	0.80
CCF-NMIBC	100 (21–100)	86 (49–97)	50 (10–91)	100 (61–100)	88 (53–98)	–	–
CCF-Combined	86 (49–97)	79 (62–89)	46 (23–71)	96 (82–99)	80 (65–90)	22.29 (2.29–216.93)	0.82
Ben-David et al.	83 (66–93)	59 (48–69)	42 (30–55)	90 (80–96)	65 (56–73)	6.84 (2.36–19.75)	0.71
Overall combined	83 (68–92)	65 (55–73)	43 (32–55)	92 (84–97)	69 (61–76)	9.13 (3.50–23.8)	0.74

ctDNA, circulating tumor DNA; CI, confidence interval; PPV positive predictive value; NPV, negative predictive value; OR, odds ratio; AUC, area under the curve; CCF, Cleveland Clinic cohort; CCF, Cleveland Clinic cohort; MIBC, muscle‐invasive bladder cancer; NMIBC, non–muscle‐invasive bladder cancer

^a^The table summarizes test performance metrics and combined estimates, demonstrating consistent high negative predictive value and reproducible ctDNA performance across cohorts

Diagnostic performance was additionally explored according to timing of ctDNA sampling relative to systemic therapy (Table S1).<ST1> The single false-negative case involved a patient sampled during neoadjuvant chemotherapy. In sensitivity analysis excluding the patients sampled during active chemotherapy, the negative predictive value increased to 100 % (95 % CI, 83–100 %), with a sensitivity of 100 % (95 % CI, 57–100 %) and an overall accuracy of 80 % (95 % CI, 63–90 %).

### External Cohort Analysis

To assess reproducibility, published data from Ben-David et al.^9^ (*n* = 109) were analyzed separately and then incorporated into a pooled descriptive analysis with the current cohort. That study included patients undergoing radical cystectomy with pelvic LND, preoperative tumor-informed ctDNA testing using the same assay platform, and complete pathologic nodal staging.

In the Ben-David cohort alone, the negative predictive value was 90 % (95 % CI, 80–96 %), with a sensitivity of 83 % (95 % CI, 66–93 %), a specificity of 59 % (95 % CI, 48–69 %), a positive predictive value of 42 % (95 % CI, 30–55 %), and an overall accuracy of 65 % (95 % CI, 56–73 %). The odds ratio for nodal metastases was 6.84 (95 % CI, 2.36–19.75), and the area under the curve was 0.71 (Table 2; Fig. 1D)

Integration of both cohorts yielded a combined population of 149 patients. In the pooled descriptive analysis, the negative predictive value was similarly high at 92 % (95 % CI, 84–97 %), with a sensitivity of 83 %, a specificity of 65 %, and an overall accuracy of 69 %. The pooled odds ratio for nodal metastases was 9.13 (95 % CI, 3.50–23.8), and the area under the curve was 0.74 (Table 2; Fig. 1D).

### Clinical Impact Analysis

A simulated ctDNA-guided strategy for our institutional cohort would have avoided pelvic LND in 27 (68 %) of the 40 patients by omitting dissection for those with ctDNA-negative results. Among these, 26 patients were correctly classified as node-negative on final pathology, whereas 1 patient had limited pN1 disease, representing a single false-negative case (2.5 % of the total cohort; 3.7 % of ctDNA-negative patients).

All the remaining ctDNA-positive patients (13 of 13) would have proceeded to LND, including six patients with node-positive disease identified at final pathology. The distribution of true- and false-negative classifications in the institutional cohort is shown in Fig. 2A, with corresponding distributions in the external and pooled cohorts displayed in Fig. 2B and C.

### Recurrence

The single false-negative case involved micropapillary muscle-invasive disease. From this patient, ctDNA was obtained 55 days before surgery during neoadjuvant chemotherapy. The ctDNA converted to positive 34 days after cystectomy, and radiographic recurrence was documented 6.5 months postoperatively.

During the follow-up period, 4 (31 %) of the 13 ctDNA-positive patients experienced recurrence, predominantly at distant sites, after a median of 5.2 months. No additional recurrences were experienced by the ctDNA-negative patients.

## Discussion

In this prospective cohort of 40 patients undergoing radical cystectomy, preoperative tumor-informed ctDNA demonstrated a negative predictive value of 96 % for pathologic nodal metastases. Of the 27 (68 %) ctDNA-negative patients, 26 truly had negative nodes, and 1 had nodal metastasis (false-negative case). These findings suggest that ctDNA-negative status may identify a clinically relevant low-risk group for nodal involvement, although this observation requires prospective validation. Importantly, the observed false-negative rate of 3.7 % among the ctDNA-negative patients was below the predefined 5–10 % threshold considered clinically acceptable, indicating that the a priori safety benchmark was achieved in this cohort. Given the relatively low prevalence of nodal metastases in this cohort, the negative predictive value should be interpreted in this context because it is inherently influenced by disease prevalence.

The clinical relevance of these findings should be interpreted within the ongoing debate regarding the therapeutic value of pelvic LND. Although LND provides important staging information, randomized trials have not demonstrated a clear overall survival advantage for more extensive templates, and the procedure is associated with measurable perioperative morbidity. Given the 10 % overall complication rate associated with LND (lymphocele 8 %, lymphedema 3 %, vascular injury 2 %), such a reduction could meaningfully decrease morbidity and operative burden.^10^ In this context, a biomarker capable of identifying patients at low risk for nodal metastases may help refine the selective use of LND rather than support its routine application for all patients.

In a simulated analysis, a ctDNA-guided approach would have avoided LND for approximately two thirds of the patients while missing a single case of limited nodal disease. An important consideration in this context is the potential impact of missed nodal disease. Omission of pelvic LND may result in understaging, which could influence postoperative risk stratification and eligibility for adjuvant systemic therapy. Accurate nodal staging remains a key determinant of prognosis and guides the use of adjuvant treatments, including chemotherapy and immunotherapy. Therefore, any biomarker-guided de-escalation strategy must ensure that clinically significant nodal disease is not systematically missed, and that downstream treatment decisions are not adversely affected.

Patients with ≥cT3 disease represent a higher-risk subgroup for whom omission of pelvic LND based on ctDNA status alone may not be appropriate due to the risk of understaging. More broadly, omission of pelvic LND should not be considered outside a protocolized prospective trial setting.

Importantly, current National Comprehensive Cancer Network (NCCN) and European Association of Urology (EAU) guidelines recommend pelvic LND at the time of radical cystectomy based on staging benefit and potential therapeutic value. The current findings do not challenge these recommendations, but instead suggest that biomarker-informed refinement of surgical extent could be explored in future prospective trials.

Future approaches may benefit from integrating ctDNA with established clinical and radiographic variables to improve risk stratification. Conventional imaging alone has limited sensitivity for detecting nodal metastases, and ctDNA may provide complementary biologic information reflecting systemic tumor burden. A multimodal model incorporating ctDNA status, imaging findings, and clinicopathologic features could potentially improve identification of patients at low versus high risk of nodal involvement compared with any single method.

Pathologic complete response was observed in 16 patients (40 %) overall, including 15 patients who were ctDNA-negative. More than half of the ctDNA-negative patients with muscle-invasive disease achieved pT0N0 status at cystectomy. This observation may reflect lower overall tumor burden, but it also may have been influenced by the effects of neoadjuvant therapy on ctDNA detectability.

Discordant cases may reflect tumor heterogeneity, including variants not captured in the initial TURBT specimen or the emergence of resistant clones after neoadjuvant therapy, which may affect ctDNA detectability.

Although preoperative tumor-informed ctDNA demonstrated high negative predictive value, performance may be influenced by sampling timing and systemic therapy exposure. The only discordant case occurred when ctDNA was obtained during neoadjuvant chemotherapy, suggesting that treatment may affect detectability. In sensitivity analysis excluding the patients sampled during active chemotherapy, negative predictive value increased to 100 %, further supporting the importance of standardized ctDNA sampling windows in future studies. For safety, ctDNA assessment could be standardized at the diagnosis of muscle-invasive disease and again after completion of systemic therapy in future studies. In an independent external cohort using the same tumor-informed assay platform, comparable diagnostic performance and preserved negative predictive value were observed, supporting the reproducibility of these findings.

Prospective validation of this approach will require carefully designed clinical trials. Key elements of an ideal study would include a homogeneous patient population, preferably restricted to muscle-invasive disease, standardized timing of ctDNA sampling relative to systemic therapy, and predefined safety thresholds for acceptable false-negative rates. Endpoints should include not only diagnostic performance but also oncologic outcomes such as recurrence patterns and survival, as well as the impact on surgical morbidity. Such trials will be essential to determine whether biomarker-guided refinement of pelvic LND can be safely implemented.

Several limitations of this study should be acknowledged. The sample size was modest, resulting in wide confidence intervals, particularly for sensitivity estimates. Follow-up evaluation was limited, and recurrence findings remain exploratory. The clinical impact analysis was simulated rather than prospectively implemented. To analyze ctDNA, the study used a binary classification, and evaluation as a continuous variable, which may provide additional prognostic resolution, was not performed. In addition, variability in neoadjuvant therapy exposure and timing of ctDNA sampling may have influenced performance estimates. The pooled analysis was based on aggregated ctDNA-stratified contingency table data and should be interpreted as a descriptive reproducibility assessment rather than formal external validation. Because individual patient data were not available, adjustment for differences in disease stage, neoadjuvant therapy exposure, and ctDNA sampling timing between cohorts was not possible. These factors may influence diagnostic performance estimates, and the pooled results should therefore be interpreted cautiously and considered hypothesis-generating.

## Conclusion

Preoperative tumor-informed ctDNA demonstrated a high negative predictive value for pathologic nodal metastases at radical cystectomy. Circulating tumor DNA-negative status identified a substantial subset of patients at low risk for nodal involvement. These findings support prospective validation and may inform the design of clinical trials evaluating biomarker-guided refinement of pelvic LND. Larger multicenter studies with standardized sampling protocols and longer follow-up periods are required before clinical implementation.

## Supplementary Information

Below is the link to the electronic supplementary material.

Supplementary file1 (DOCX 16 kb)

## References

[1] van der Heijden AG, H.M.B., Carrion A, Cathomas R, Compérat EM, Dimitropoulos K, Efstathiou JA, et al., *EAU Guidelines on Muscle-invasive and Metastatic Bladder Cancer*. EAU Guidelines Office, Arnhem, 2025.

[2] National Comprehensive Cancer Network. *NCCN Clinical Practise Guidelines in Oncology (NCCN Guidelines), Bladder Cancer* (version 1.2025). 2025 17.09.2025. Retrieved xxxx at https://www.nccn.org/professionals/physician_gls/pdf/bladder.pdf.

[3] Heck MM, et al. Long-term results from the LEA randomized trial: extended versus standard lymph node dissection in patients with bladder cancer undergoing radical cystectomy. *Eur Urol*. 2025;88:566–70. 40967930 10.1016/j.eururo.2025.09.001

[4] Lerner SP, et al. Standard or extended lymphadenectomy for muscle-invasive bladder cancer. *N Engl J Med*. 2024;391:1206–16. 39589370 10.1056/NEJMoa2401497 PMC11599768

[5] Christensen E, et al. Early detection of metastatic relapse and monitoring of therapeutic efficacy by ultra-deep sequencing of plasma cell-free DNA in patients with urothelial bladder carcinoma. *J Clin Oncol*. 2019;37:1547–57. 31059311 10.1200/JCO.18.02052

[6] Wang B, et al. Real-world experience with a commercial circulating tumor DNA assay in non–muscle-invasive bladder cancer. *Eur Urol Oncol*. 2025;8:883–7. 40517059 10.1016/j.euo.2025.05.019

[7] Chatzkel J, et al. Urothelial cancer: leveraging circulating tumor DNA to avoid unnecessary bladder-directed and systemic therapies. *Eur Urol Focus.* 10.1016/j.euf.2025.07.012 40716995

[8] Tasios A, et al. In patients with muscle-invasive bladder cancer undergoing radical cystectomy, dynamics of circulating tumor DNA following cystectomy: association with patient outcomes. *Eur Urol Focus.* 10.1016/j.euf.2025.06.018 40651904

[9] Ben-David R, et al. Longitudinal tumor-informed circulating tumor DNA status predicts disease upstaging and poor prognosis for patients undergoing radical cystectomy. *Eur Urol Oncol*. 2024;7:1105–12. 38521660 10.1016/j.euo.2024.03.002

[10] Gschwend JE, et al. Extended versus limited lymph node dissection in bladder cancer patients undergoing radical cystectomy: survival results from a prospective, randomized trial. *Eur Urol*. 2019;75:604–11. 30337060 10.1016/j.eururo.2018.09.047

