# Supplementary material for: Preoperative Tumor-Informed ctDNA for Prediction of Nodal Metastases at Radical Cystectomy in Bladder Cancer
Source: Ann Surg Oncol. 2026 Jun 21;33(10):9475–82. doi: 10.1245/s10434-026-20022-7 (PMC13585835; doi:10.1245/s10434-026-20022-7)
Supplement: Supplementary file 1 [file 10434_2026_20022_MOESM1_ESM.docx]

| **Sampling timing** | **n** | **Sensitivity** | **Specificity** | **PPV** | **NPV** | **Accuracy** | **AUC** |
| --- | --- | --- | --- | --- | --- | --- | --- |
| Before NAC | 3 | NE | 100 (44–100) | NE | 100 (44–100) | 100 (44–100) | NE |
| During NAC | 10 | 50 (9–91) | 88 (53–98) | 50 (9–91) | 88 (53–98) | 80 (49–94) | 0.69 (0.23–1.00) |
| After NAC | 12 | 100 (34–100) | 80 (49–94) | 50 (15–85) | 100 (68–100) | 83 (55–95) | 0.90 (0.60–1.00) |
| Without NAC | 15 | 100 (44–100) | 67 (39–86) | 43 (16–75) | 100 (68–100) | 73 (48–89) | 0.83 (0.53–1.00) |
| All patients but excluding those sampled during NAC | 30 | 100 (57–100) | 76 (57–89) | 45 (21–72) | 100 (83–100) | 80 (63–90) | 0.88 (0.79–0.97) |

**Table S1.** Diagnostic performance of preoperative tumor-informed ctDNA for prediction of pathological nodal metastases stratified by timing of ctDNA sampling relative to chemotherapy, including a sensitivity analysis excluding patients sampled during active chemotherapy.
